# The Role of Mobility in Intertidal Invertebrates’ Responses to Thermal Stress

**DOI:** 10.1093/icb/icaf078

**Published:** 2025-06-03

**Authors:** L C McIntire, L P Miller

**Affiliations:** Coastal and Marine Institute, San Diego State University, 4165 Spruance Rd, San Diego, CA 92101, USA; Department of Evolution and Ecology, University of California, Davis, One Shields Ave, Davis, CA 95616, USA; Coastal and Marine Institute, San Diego State University, 4165 Spruance Rd, San Diego, CA 92101, USA

## Abstract

As climate change progresses, it is important to be able to predict how the effects of elevated temperatures are affected by the ability of ectotherms to seek shelter. Many studies on ectotherms have suggested that mobility is a vital characteristic to understand how species will react to warming. Highly mobile ectotherms are not often exposed to thermally stressful conditions because they can actively select temperatures that are thermally beneficial or benign. Slow-moving or sessile ectotherms, however, are not able to change habitats quickly enough to escape from thermal stress or even death. In order to measure how mobility affected how organisms cope with temperature, we quantified the body temperatures, environmental temperatures (using biomimetic models), and thermal limits using respirometry of eight intertidal ectotherms in four mobility classes: fast, intermediate, slow, and sessile. In addition, we also calculated thermal safety margins (TSMs) for each of our species. While we predicted that fast and intermediately mobile species would have lower thermal limits and narrower TSMs than slow and sessile animals, we found that faster organisms had lower thermal limits and narrower thermal safety margins than the other three mobility classes. Our findings indicate that there is an effect of mobility on how organisms cope with temperatures and lay the groundwork for understanding how communities may respond to climate change.

## Introduction

Ectothermic animals are particularly susceptible to elevated temperatures caused by climate change since they rely on their environment to moderate their body temperatures (Jørgensen et al. 2022). On a physiological level, temperature can affect metabolic rates (Sinclair et al. 2016), heat shock protein production (Dong et al. 2022), and other processes. Behaviorally, organisms may select thermally favorable habitats that either shelter them from thermal stress or help them warm their bodies. The way organisms select habitats, however, may be dependent on their mobility—defined as the capacity for movement (Crickenberger et al. 2020). How mobility affects habitat selection could potentially buffer, or exacerbate, the effects of climate change since organisms that are able to primarily rely on selecting a thermally safe habitat quickly are not exposed to selection for increased physiological responses to temperature (Huey et al. 2012). For example, lizards (*Anolis cristatellus*) have been found to have the same body temperatures and physiological thermal limits regardless of ambient temperatures (Huey et al. 2003), because they situate themselves in thermally suitable microsites. However, currently there has been no comprehensive comparison of how relative mobility affects thermoregulatory behavior, though studies have indicated that it is an important metric to understand (Huey and Tewksbury 2009). A comparison of physiology and behavior across species of varying mobility would lay the groundwork for understanding how species and communities could be affected by a warming climate.

Generally, moderate increases in temperature above normal, ambient conditions are not necessarily immediately stressful for ectotherms, since small increases in temperature may increase organismal performance (Huey and Kingsolver 1989). Performance will approach an optimal temperature where the organism functions well, but beyond that point, performance will decline and, eventually, at higher temperatures the organism will die—this rise and fall in performance has been termed a thermal performance curve (TPC, Huey and Kingsolver 1989). For tolerating larger increases in environmental temperatures, the role of avoiding high temperatures via mobility versus tolerating high temperature via physiological mechanisms is particularly important in cases where organisms are experiencing sublethal or lethal stress.

The rocky intertidal zone is a thermally dynamic environment that oscillates between relatively stable ocean temperatures at high tide and variable low-tide temperatures when animals are exposed to air temperatures that can fluctuate up to 20⁰C in just a few hours (Helmuth et al. 2011). The habitat complexity of the rocky shore often creates thermal heterogeneity that varies by microhabitat and this small-scale variation can be as great as the variation across many degrees of latitude (Denny et al. 2011; Jurgens and Gaylord 2018). Further, organisms of varying mobility within these habitats react differently to thermal stress during low tide. For example, faster organisms such as the shore crab *Hemigrapsus nudus* will shuttle between warm and cool habitats to keep their body temperatures low (McGaw 2003). Conversely, the slower sea star *Pisaster ochraceus* must select cooler habitats to avoid its thermal limits (Monaco et al. 2016). Even slower species, such as limpets, will lift their shells off the substrate and “mushroom,” which can reduce their body temperatures (Williams et al. 2005). This range of thermal stress provides an ideal system for understanding how organisms of different mobilities will be affected by climate change.

Our study aimed to quantify the thermal niches of eight intertidal species that represent different mobilities: ([fast]: *Pachygrapsus crassipes* and *Ligia occidentalis*; [intermediate]: *Tegula funebralis* and *Nucella ostrina*; [slow]: *Lottia scabra* and *L. digitalis*; and [sessile]: *Balanus glandula* and *Mytilus californianus*). While our study species are not phylogenetically independent, this comparison allows us to quantify thermal niches of co-occurring organisms under current conditions and begin to predict how warming could affect them.

We estimated thermal niches using (1) live animal body temperatures (*T*_b_); (2) operative (or environmental) temperatures (*T*_e_) from species-specific biomimetic models; (3) physiological thermal limits; and (4) thermal safety margins (TSMs) calculated from thermal limits and *T*_e_. A TSM, expressed as the difference between a species’ thermal tolerance and the extreme operative temperatures of their environment, provides a metric to estimate the vulnerability of organisms to both high and low temperatures within their environment (Sunday et al. 2014). *T*_e_ is typically measured with biomimetic models, which mimic the heating and cooling properties of the live organisms (Helmuth et al. 2011). These physical models, in conjunction with laboratory-measured physiological responses to temperature, can help quantify species’ TSMs and, therefore, potential vulnerability to warming within habitats under climate change (Helmuth et al. 2011). We predicted that (1) fast and intermediate species would be selecting cooler habitats and have lower *T*_b_ than slow and sessile species; (2) fast and intermediate species would have lower physiological thermal limits than slow and sessile organisms; and (3) the TSMs of fast and intermediate organisms would be narrower than those of the slow and sessile species.

## Methods

### Field sites

We conducted field surveys at two sites on the Bodega Marine Reserve (BMR; Bodega Bay, CA, USA, 38.31⁰N, 123.07⁰W). The intertidal zone at BMR is composed of granite benches that face predominantly west. During the summer, it experiences northwesterly waves and semidiurnal mixed tides with the lowest low occurring in the early mornings. We set up two 40-m transects, one on the north bench and one in Horseshoe Cove, in the upper intertidal zone at shore heights between +1.52 and 2.13 m above mean lower low water (MLLW).

#### Habitat surveys

We quantified the availability of habitat types (vertical surface, horizontal surface, pools, and crevices) within 1 m of the transect using a 0.25 m^2^ quadrat to quantify the % coverage along each transect (*n* = 160 quadrats/site).

#### Body temperature surveys

Surveys were carried out in the late spring to late summer (June–August) from 2021 to 2024 during daytime low tides lower than +1 m above MLLW that occurred after sunrise (2021: *n* = 7; 2022: *n* = 15; 2023: *n* = 16; and 2024: *n* = 7). We quantified *T*_b_ and habitat selection of our study species within half a meter of the transect within 3 h of the low tide.

For mobile mollusc species, we measured *T*_b_ within randomly placed 0.25 m^2^ quadrats by inserting a thermocouple wire gently between their shell and mantle. For barnacles and mussels, valves were gently parted, and the thermocouple wire was inserted inside. For the faster species, we did *T*_b_ surveys immediately upon arrival at the site as an area search within 1 m of the transect since their habitat selection would change in the researcher’s presence. *Ligia occidentalis* was measured by gently placing a thermocouple wire on the top of the carapace, verified by initial comparisons of temperatures taken internally versus externally (see Supplemental 1). *Pachygrapsus crassipes* would retreat deep into crevices if disturbed, so we used a thermal imaging camera (FLIR Systems, Model TG267, Goleta, CA, USA; emissivity = 0.97) with a ×2 lens so we could measure crab *T*_b_ from a distance, after making pilot comparisons of internal body temperature versus external temperatures (Supplement S1).

#### Biomimetic models (*T*_e_)

We created species-specific biomimetic models of all eight species to quantify the range of *T*_e_ in the field. All biomimetic models were created using resin epoxy and shells of live organisms. They were on average within 1–2°C of live organisms (for methodological details, see Supplement 2). The mimics were placed haphazardly throughout the intertidal zone in sun-exposed and sheltered habitats for the duration of the low tide and temperatures were recorded on a thermocouple data logger every 5 s.

#### Respiration rate measurements

In the laboratory, we quantified respiration rates in air for *P. crassipes, Li. occidentalis, T. funebralis*, and *N. ostrina* with closed chamber respirometry. Aerial respiration rates for the remaining four species were taken from the literature. Organisms were captured from the field and placed in a flow-through aquarium held at 12°C (monitored with Onset, HOBO TidbiT Temperature Logger, Bourne, MA, USA) for at least 1 h before being tested to give them time to recover from handling stress. All organisms were tested within 72 h of capture.

The respiration chambers were aluminum containers (volume = 52.3 mL) with a paper towel (4 cm^2^) soaked with 2 mL of seawater to keep humidity near 100%. For smaller organisms, we used resin blocks to reduce the volume of air in the chamber so they would be able to measurably reduce oxygen in the chambers (volume = 8–35 mL). We submerged the respiration chambers in water baths (ThermoFisher Scientific, Waltham, MA, USA) at 14.5°C, which were then heated at a rate of 8°C h^−1^ to the treatment temperature. Chambers had ports that were left open during the ramping period to allow air exchange and closed once the treatment temperature was reached, followed by a 1-h sampling period where oxygen sensor spots (Pt3, PreSens, Regensburg, Germany) placed inside the chamber were sampled using fiber optic cables attached to a data logger (oxy-4, PreSens). Twelve individuals per species were exposed to each temperature treatment (∼14.5, 26, 32°C), and each individual was only used in one trial. Temperatures were selected starting with the average air temperatures at BMR during summertime morning low tides (14.5°C, L. M. McIntire, unpublished) and increased to be below previously published limits for either closely related species or their thermal limits in water (McGaw 2003; Eberl et al. 2013; Gleason and Burton 2013; Hayford et al. 2018). An empty chamber was included in each trial to serve as a control for potential sensor drift, and sensors were calibrated using a two-point calibration process at 0% O_2_ using nitrogen gas, and water-saturated air for normoxia.

#### Thermal limits (LT_50_s) and thermal safety margins (TSMs)

We monitored mortality during respirometry trials to determine the temperature at which 50% mortality (LT_50_) was achieved. Organisms would be removed from the chambers and observed for 30 min, and if no response was elicited by gentle probing, the organism was considered dead. If 100% mortality was not achieved, we then would increase the temperature treatment until complete mortality occurred ([*P. crassipes*: 35.0, 36.9, 37.4°C]; [*Li. occidentalis*: 36.7 and 38.6°C]; [*T. funebralis:* 40.6 and 44°C]; and [*N. ostrina*: 37.1 and 39.1°C]). We used published LT_50_ values for *L. digitalis* (Dong and Somero 2009), *M. californianus* (Jurgens and Gaylord 2016), and *B. glandula* (Gilman et al. 2015). While *L. scabra* LT_50_ measurements were unavailable in the literature, Miller et al. (2015) measured the critical maximum temperature (39.6°C, CT_max_) as the temperature where locomotor function was lost (Orsted et al. 2022).

We calculated the TSMs by subtracting the 99th percentile of *T*_e_ observed for biomimics of each species and that species’ thermal limits (LT_50_ or CT_max_). This allowed us to encompass the most stressful conditions during a typical northern California summer. Negative numbers indicate that a habitat is not thermally safe for organisms, and they may need to avoid stressful *T*_b_, while positive values indicate that temperatures typically do not exceed species’ thermal limits (Sunday et al. 2014).

#### Data analysis

We calculated the frequency at which *T*_b_ and *T*_e_ values were within a range of stressful temperatures and at/above the thermal limits in the field. Stressful temperatures were defined based on optimal temperatures from TPCs (see below). Differences in *T*_b_ between species were tested using ANOVA and the post-hoc Tukey test. To compare *T*_b_ across mobility groups, we used a hierarchical model with species as a random factor.

Respiration rates were calculated using the slopes of the best-fit least-squares regression line between elapsed time and oxygen concentration, and rates were compared within species using ANOVA. The data violated the assumption of homogeneity of variances, so we used the nlme package to weigh each average proportional to its variance (Pinheiro et al. 2025) in R (version 4.1.2, R Core Team 2021). Post-hoc Tukey comparisons were made using the multcomp package (Hothorn et al. 2008). Optimal temperatures were determined by fitting a TPC to the respiration data (Sharpe-Schoolfield; Smith et al. 2019) using the package rTPC (Padfield et al. 2025). For literature sources, we used ImageJ (Schneider et al. 2012) to estimate the peak of their respiration rates. LT_50_ values were calculated by fitting a curve to the mortality data in the MASS package (Venables and Ripley 2002).

## Results

### Respirometry and LT_50_

**Fig. 1:**
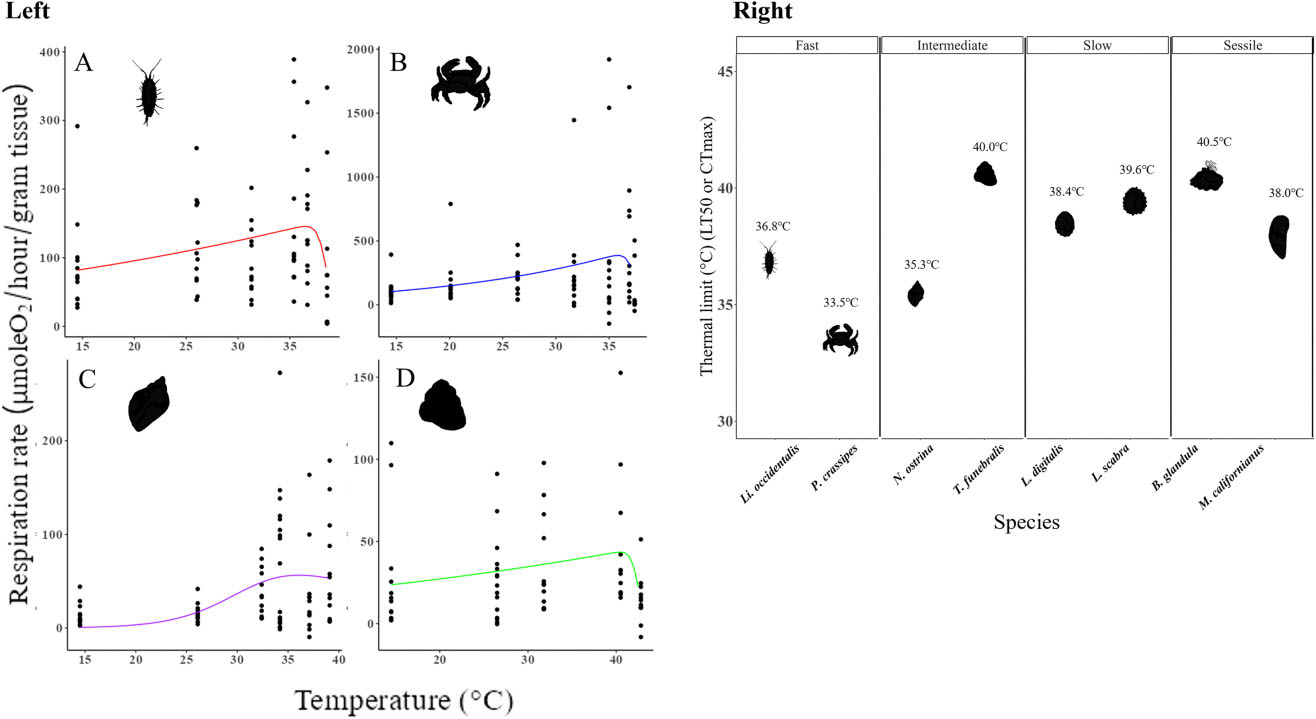
Left panel: Mass-adjusted mean respiration TPCs for (A) *Li. occidentalis*, (B) *P. crassipes*, (C) *N. ostrina*, and (D) *T. funebralis*. Right panel: thermal limits (LT_50_, CT_max_) of all species (*Li. occidentalis; P. crassipes; T. funebralis; N. ostrina* [all LT_50_, this study]; *L. scabra* [CT_max_, Miller et al. 2015]; *L. digitalis* [LT_50_, Dong and Somero 2009]; *B. glandula* [LT_50_, Ober et al. 2019]; *M. californianus* [LT_50_, Monaco et al. 2016]).

Among the fast-moving pair of species, *Li. occidentalis* and *P. crassipes*, there were no significant differences in respiration rate between temperature treatments. For *Li. occidentalis*, respiration rates were low at 14.5°C and their respiration rates peaked at 36.6°C, while at higher temperatures (39.1°C) their respiration rates declined, and they began to die (Fig. 1; LT_50_ = 36.8°C, nonsignificant ANOVA results are shown in Supplement 3). *Pachygrapsus crassipes* consumed less oxygen between 14.5 and 20.1°C but peaked at 35.8°C (Fig. 1) and had an LT_50_ = 33.5°C (Fig. 1). Their respiration peaked above their LT_50_ since they died over a wide range of temperatures.

For the intermediate pairing, *T. funebralis* respiration rates peaked at 40.6°C (Fig. 1; LT_50_ = 40°C), though temperature treatments were not significantly different from each other (Supplemental 3). *Nucella ostrina* respiration rates were statistically different from each other between lower and higher temperatures (Fig. 1; Supplemental 3; ANOVA; *F*_4,61_ = 6.12; *P* < 0.01). At 14.5 and 26.1°C, their respiration rates were similar (Tukey; *Z*_4,11_ = 0.43; *P* = 0.99), but respiration rates increased significantly at higher temperatures between 32.5 and 34.2°C, peaking at 36.1°C (Fig. 1; LT_50_ = 35.3°C).

We took slow species respiration and thermal limits from the literature. *Lottia scabra* respiration rates peaked at 35°C and their CT_max_ was 39.6°C (Fig. 1; Miller et al. 2015). *Lottia digitalis* respiration rates peaked at 35°C (Bjelde and Todgham 2013) and their LT_50_ was measured at 38.4°C (Fig. 1; Dong and Somero 2009).

Sessile species’ thermal performances were also taken from the literature. *Mytilus californianus* respiration rates peaked at 27°C (Monaco et al. 2016) and their LT_50_ was 38°C (Fig. 1; Jurgens and Gaylord 2016). For *B. glandula*, respiration rates were highest at 30°C (Ober et al. 2019) and their LT_50_ was 40.5°C (Fig. 1; Gilman et al. 2015).

### *T*_b_ and *T*_e_

**Fig. 2:**
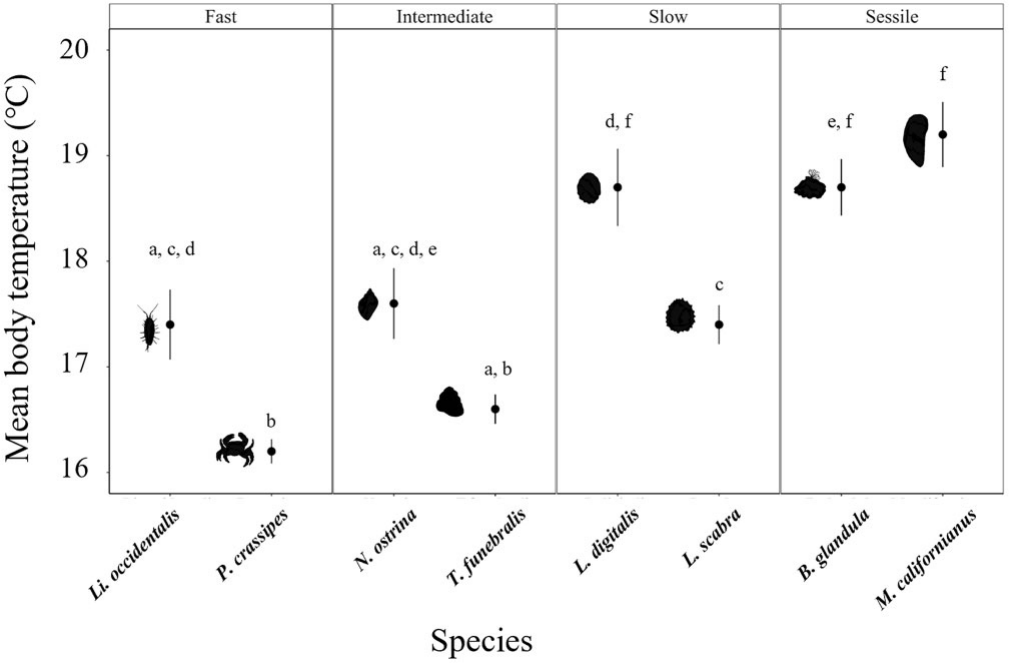
Average *T*_b_ of species from field surveys during low tide. There was no statistical difference between the mobility classes (ANOVA; *F*_3,2278_ = 4.2; *P* = 0.10), but there was a difference between species within those classes (ANOVA; *F*_7,2274_ = 22.05; *P* ≪ 0.01). Letters represent statistical similarity. Bars represent standard errors.

**Fig. 3:**
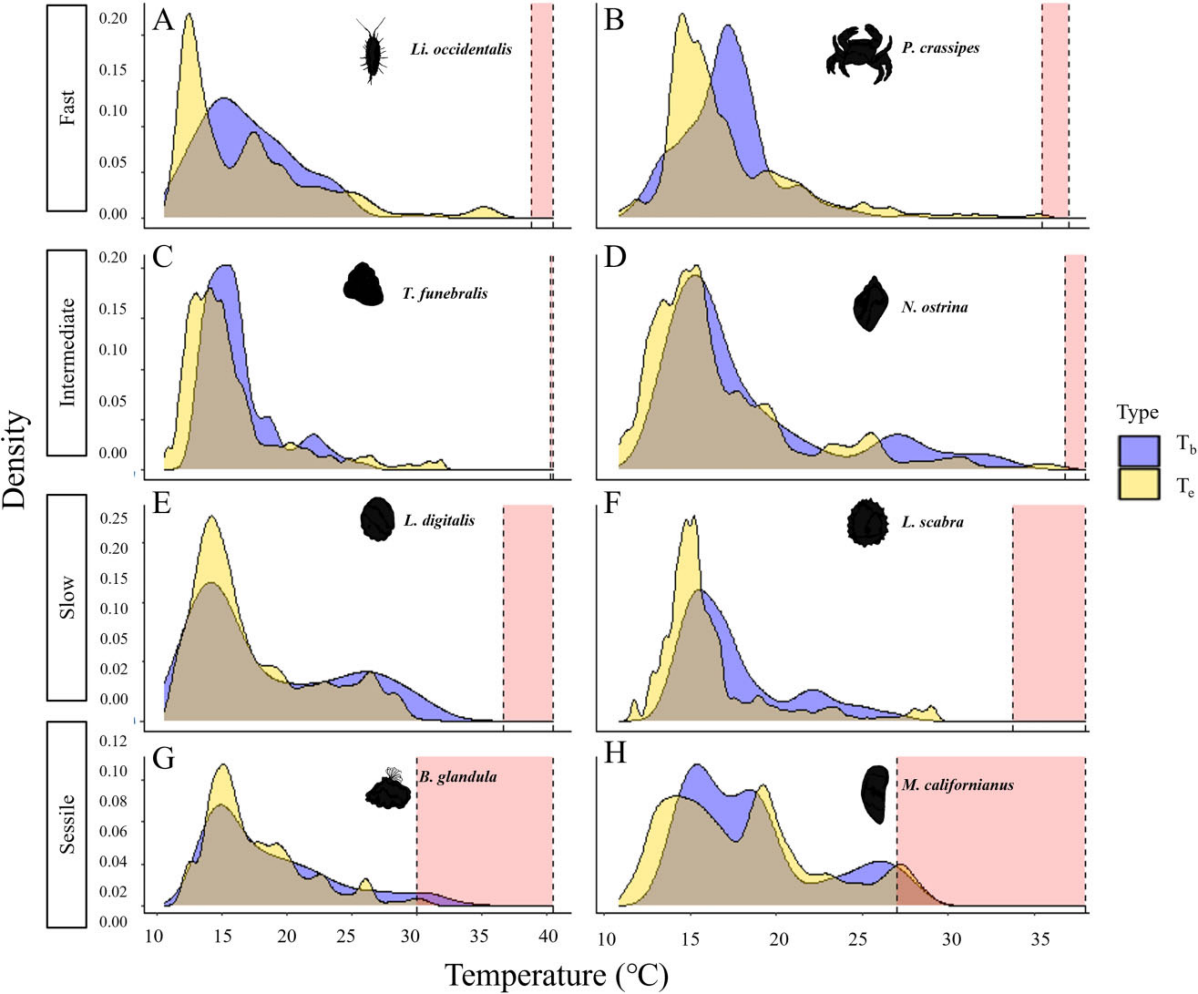
Densities of animal body temperatures (*T*_b_) and operative temperatures of biomimics (*T*_e_) based on field measurements during daytime low tides. The left edge of each shaded region is the temperature at which the species’ respiration rate peaked, while the right edge denotes the species’ thermal limits from this study or the indicated reference: (A) *Li. occidentalis*; (B) *P. crassipes*; (C) *T. funebralis*; (D) *N. ostrina*; (E) *L. scabra* (CT_max_, Miller et al. 2015); (F) *L. digitalis* (LT_50_, Dong and Somero 2009); (G) *B. glandula* (LT_50_, Ober et al. 2019); and (H) *M. californianus* (LT_50_, Monaco et al. 2016).

All species were found to nonrandomly select habitats (Supplement 4). *T*_b_ in the field did not vary significantly between mobility classes (ANOVA; *F*_3,2278_ = 4.2; *P* = 0.10), but there was still a trend toward faster species being cooler than the other mobility classes (Fig. 2). There were also differences between species within the mobility classes (Fig. 2; ANOVA; *F*_7,2274_ = 22.05; *P* ≪ 0.01). Further, all species were predominately found in cooler, nontressful temperatures (∼12–20°C; Fig. 3). However, *T*_b_ values of the fast species, *Li. occidentalis* and *P. crassipes*, were never above their thermal limits and individuals were rarely found within thermally stressful ranges (Fig. 3A and B). In the typical summer conditions at our sites, their *T*_e_ was also rarely above their thermal limits (Fig. 3A and B). Similarly, the intermediately mobile *T. funebralis* were never above their thermal limits or within their stressful range (Fig. 3C). *Nucella ostrina*, however, was within a stressful temperature range 6% of the sampling period, but never above their lethal limits (Fig. 3D). The slow-moving species, *L. scabra* and *L. digitalis*, were never in their stressful range (Fig. 3E and F). Similarly, their *T*_e_ was never above their thermal limits or within their stressful range (Fig. 3E and F). The sessile species, *B. glandula* and *M. californianus*, were never found above their thermal limits, but their *T*_b_ was within their stressful range 6 and 10% of the time and the biomimic *T*_e_ was within their stressful range 1 and 10% of the time, respectively (Fig. 3G and H).

**Fig. 4:**
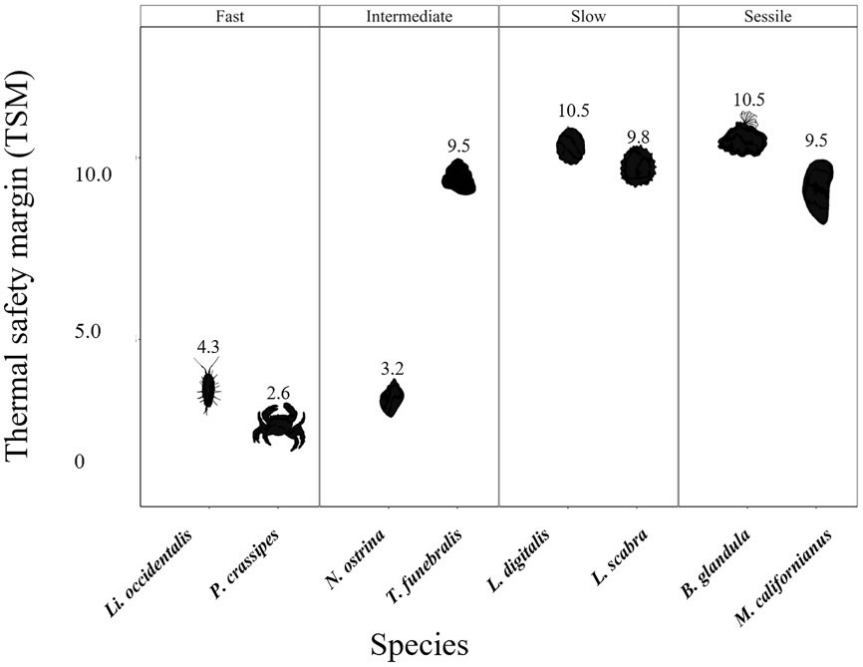
TSMs for each species, calculated by subtracting their thermal limits (CT_max_ or LT_50_) from the 99th percentile of *T*_e_ in the field (*Li. occidentalis; P. crassipes; T. funebralis; N. ostrina* [all LT_50_, this study]; *L. scabra* [CT_max_, Miller et al. 2015]*; L. digitalis* [LT_50_, Dong and Somero 2009]*; B. glandula* [LT_50_, Ober et al. 2019]; and *M. californianus* [LT_50_, Monaco et al. 2016]).

TSMs were smallest for *P. crassipes*, and *Li. occidentalis* and *N. ostrina* both had small TSMs (Fig. 4). While *T. funebralis* has a higher capacity for movement, it has a comparatively high thermal limit (Fig. 1) and TSM (Fig. 4). *Lottia scabra, L. digitalis, M. californianus*, and *B. glandula* all had relatively larger positive TSMs because of their high thermal limits; therefore, they experience fewer risky days than their faster counterparts during a typical Northern California summer (Fig. 4).

## Discussion

Overall, thermal limits and TSMs for fast species were lower than those of intermediate, slow, and sessile species, except for *N. ostrina*. We had predicted that fast and intermediate species would be similar but found that *T. funebralis* was more similar to slow and sessile species. Slow and sessile species’ wider thermal niches are potentially a consequence of their reliance on cellular-level and organ-system-level physiological responses rather than behavior, while faster species are avoiding thermal stress, resulting in a narrow thermal tolerance niche (Buckley et al. 2015). Like our fast-moving crustacean species, highly mobile crabs in the genus *Petrolisthes* that live in cooler microhabitats than our study species also have narrow TSMs (Stillman and Somero 2000). Consequently, less-mobile organisms are potentially less susceptible to increased temperatures since they have higher thermal limits. Conversely, highly mobile organisms may be at higher risk under climate warming since they are unable to physiologically cope with elevated temperatures if thermal refuges are unavailable (Buckley et al. 2015). Our data were collected under typical northern California summer conditions, but TSMs could be narrower under extreme heatwave events that can occur in northern California, particularly in the spring when the lowest tides are during midday (Harley 2008). Further, the effects of a mosaic of microhabitat warming could also be a factor (Woods et al. 2015). For example, crevices may warm less than horizontal surfaces; however, for species like our fast species, even a few degrees of warming could be detrimental and could relegate them to only cooler microhabitats.

It is important to acknowledge that the study species we chose necessarily involved a phylogenetic confound, as the fast-moving species are both crustaceans, while the intermediate and slow-moving species are all gastropods. This leads to morphological differences that are important for thermal relations (i.e., warming from contact with substrate by molluscan foot versus crustacean legs or the energy expense of molluscs creating a mucus layer to move). Our study design is driven by the existence of these co-occurring species as members of a present-day ecological community that experience the same set of site-level weather conditions, while potentially experiencing different species-specific thermal histories driven by their mobility or physiological tolerance.

For example, *Li. occidentalis* and *P. crassipes* can move fast enough to make instantaneous decisions to find shelter from elevated temperatures, but molluscs, even the intermediate species such as *T. funebralis* and *N. ostrina *, cannot. Slow-moving organisms such as*L. scabra* and *L. digitalis* also cannot move quickly enough to protect themselves from stressful temperatures during a single low tide—which could prove lethal (Dong et al. 2008, 2009). For these species, making a “mistake” when selecting habitats at the start of a low tide period, or even over the course of days or weeks, is potentially more detrimental (Hui et al. 2022). Sessile animals such as *B. glandula* and *M. californianus* experience heavy selection for thermal tolerance after settlement due to thermal stress (Logan et al. 2012; Ober et al. 2019, respectively). These consequences could result in the selection for less-mobile animals having higher thermal limits than faster species since fast animals rely on behavior at the expense of physiological responses (Huey et al. 2012).

The variation in thermal limits (Fig. 1) and TSMs (Fig. 4) between mobility classes may not be solely due to their ability to quickly move in direct response to thermal stress, but instead due to their behavior during high tide. For example, *N. ostrina* has been documented to have a circatidal rhythm by which they will change their position on the shore each day before the low tide throughout the 2-week tidal cycle (Hayford et al. 2018). Therefore, while *N. ostrina* was classified in this study as an organism with intermediate mobility, it is selecting thermally benign habitats during the high tides (Hayford et al. 2018). This has also been demonstrated in other slow-moving species, such as *Pisaster ochraceus*, which will increase its internal water volume and move lower on the shore after thermally stressful low tides, which allows them to increase their thermal inertia (Pincebourde et al. 2009). This type of behavior has been termed “bet hedging” and has been documented in many slow-moving intertidal ectotherms (Pincebourde et al. 2009; McIntire and Bourdeau 2020).

Like the difference in TSM observed between *T. funebralis* and *N. ostrina* that may be linked to their temperature stress avoidance strategy, the slow-moving limpets *L. scabra* and *L. digitalis* also differ in their TSM and movement patterns. The homing behavior of *L. scabra* limits its location on the shore, because it must be able to forage and return to its home scar before the tide falls again, meaning that it is effectively less mobile than some of its congeners (Wolcott 1973). Conversely, *L. digitalis* exhibits an aggregating behavior that allows it to seek out habitats more opportunistically and will shift their aggregations away from “hot spots” (Frank 1964). This difference in behavior has been hypothesized to be the reason why these organisms have different thermal tolerances (Wolcott 1973; Dong et al. 2008). It is worth noting that it is generally well documented that *L. scabra* has a higher thermal limit than *L. digitalis* (Wolcott 1973; Dong et al. 2008); however, in our study, we did find that they were on average cooler than *L. digitalis*. This is likely due to the nature of the temperature sampling, particularly in the early morning when some substrates were not yet exposed to the sun. Overall, for slower-moving organisms such as molluscs, it is important to consider not only instantaneous behavior, but also behavior that is taking place during nonstressful high tides.

Further, some species may be selecting habitats for other reasons, such as access to food and mates, or avoiding predators. For example, fiddler crabs will traverse hot, dry habitats to access mates (Allen and Levinton 2014), and geckos will choose to avoid predators over accessing thermally beneficial habitats (Downes and Shine 1998). Additional studies measuring how individuals select thermal habitats when exposed to predators or to find food are needed to understand the tradeoffs organisms are making when selecting habitats.

Our methods for determining thermal limits in the laboratory removed other abiotic factors such as humidity, wind, and solar radiation. Consequently, the thermal limits in our study are likely higher than what an organism would be able to handle in the wild since they may often be dealing with desiccation stress in addition to thermal stress (Iacarella and Helmuth 2011). Additionally, we determined mortality within 30 min of temperature exposure, so our LT_50_ may be slightly less conservative than the other studies cited for thermal limits of our slow and sessile species (Kingsolver and Woods 2016). Studies that evaluate survival after longer periods (hours to days) may find mortality at slightly lower peak temperatures, which would decrease our species TSMs further, thus creating the same pattern of fast species having lower thermal limits than the other species in our study.

Understanding how mobility affects the susceptibility of organisms to climate change is vital for understanding the ecological consequences of elevated temperatures. In our study, highly mobile organisms had narrower TSMs, which are likely to be exacerbated by climate change. Additionally, slower and sessile organisms may have reduced TSMs in the future. Not only are they going to be potentially experiencing more frequent lethal stress events, but sessile organisms are experiencing higher temperatures, which could result in higher levels of energetic need during low tides. There is also the possibility that these organisms will no longer be able to rely on their movement behaviors to avoid thermal stress as temperatures continue to increase and thermal refuges become less available, and so quantifying operative environmental temperatures and thermal safety margins now will help give insight into how these communities may respond to climate change.

## Author contributions

L.C.M. and L.P.M. conceived of the presented idea. L.C.M. carried out data collection. Both authors analyzed and discussed the results and contributed to the final manuscript.

## Supplementary Material

icaf078_Supplemental_Files

## Data Availability

Data accompanying this manuscript are made available at https://doi.org/10.5281/zenodo.15400631
