# Supplementary material for: The Role of Mobility in Intertidal Invertebrates’ Responses to Thermal Stress
Source: Integr Comp Biol. 2025 Jun 3;65(4):812–21. doi: 10.1093/icb/icaf078 (PMC12530186; doi:10.1093/icb/icaf078)
Supplement: icaf078_Supplemental_Files [file icaf078_supplemental_files.zip › icb-2025-0113-File007.docx]

*Supplemental material for* McIntire LM & Miller LP. The role of mobility in intertidal invertebrates’ responses to thermal stress

Supplement 1: Comparison of the internal and external body temperatures of fast species

Since we could not insert wires non-lethally into the body cavities of the isopod *Li. occidentals* and *P. crassipes* we did an initial comparison of their internal body temperatures and external carapace temperatures by inserting a thermocouple wire into their body cavity and gluing (Loctite™, Westlake, OH, USA) a thermocouple wire (type K, 36 gauge attached to an Omega HH508, Norwalk, CT, USA) to their carapace in the field (n = 8/species; *Li. occidentalis* mean difference: 0.47 ± 0.02℃; *P. crassipes* mean difference: 0.85 ± 0.22℃).

Supplement 2: Mimic building and validation

We used resin as it is a common filler for biomimetic models since it has similar thermal characteristics to organismal tissue (Hayford et al. 2021; PuDuo Resin, USA). *Li. occidentalis* mimics were made from black resin casts of the real animals. *P. crassipes* biomimetic models were a carapace filled with resin with a water filled sponge placed on top of a resin cast of a crab to allow for evaporative cooling. *P. crassipes* size impacted how quickly thermal transfer occurred, so smaller crabs were made from black resin and larger ones were made from clear resin. All other biomimetic models were constructed from the shells of the animal and filled with resin. For the gastropod mimics, we applied a thin layer of glycerol (glycerine and hydroxyethylcellulose, KY, NY, USA) between the rock and their resin “foot” to mimic the mucus layer of a live mollusc.

All biomimetic models were validated by comparing them to live organism temperatures (see figures below). We placed live animals side-by-side with biomimetics in the laboratory and in the field with thermocouple wires inserted in each. In the laboratory, we used reptile heat lamps to capture a wide temperature range (from 12-35℃, except for *P. crassipes*, which died at 35℃, those trials stopped at 30℃). In the field we deployed biomimics and live animals for three hours after low tide and recorded temperature readings of the animals and biomimetic models every minute (n=8 /species).


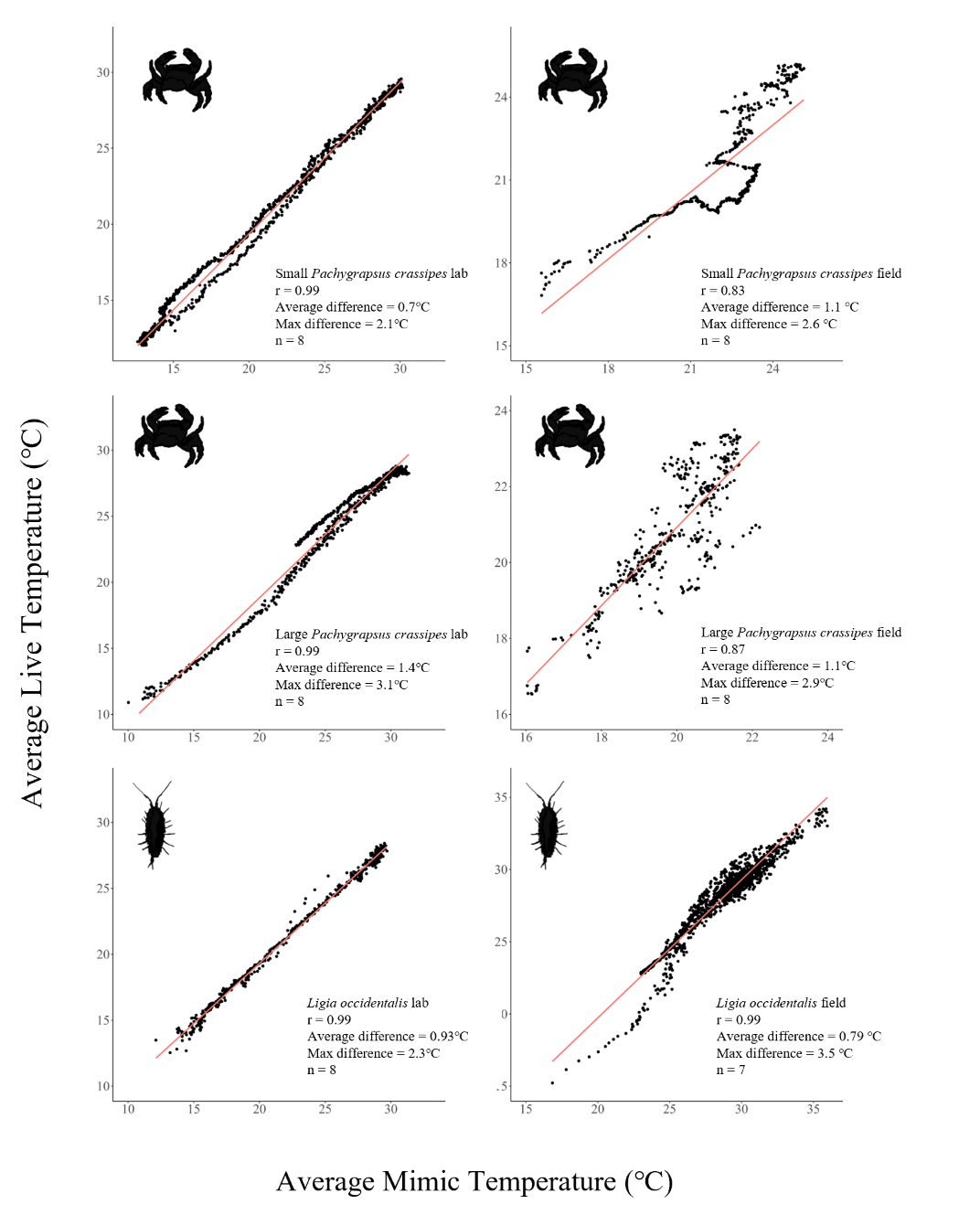


Figure S2A: The relationship between biomimic temperatures and live animal temperatures from both indoor heat lamp trials (left column) and outdoor field trials (right column) is shown for the two fast-moving species. Eight live animals and 8 mimics were placed together either in the laboratory or in the field with thermocouple wires embedded in both. Temperatures were taken every 5 seconds over the course of 2 hours in the lab and 1 hour in the field. Points represent the average temperature of 8 mimics and 8 live animals at a given time point. The red line indicates a 1:1 relationship, so points falling below the line indicate that the biomimic was cooler than the live animal.


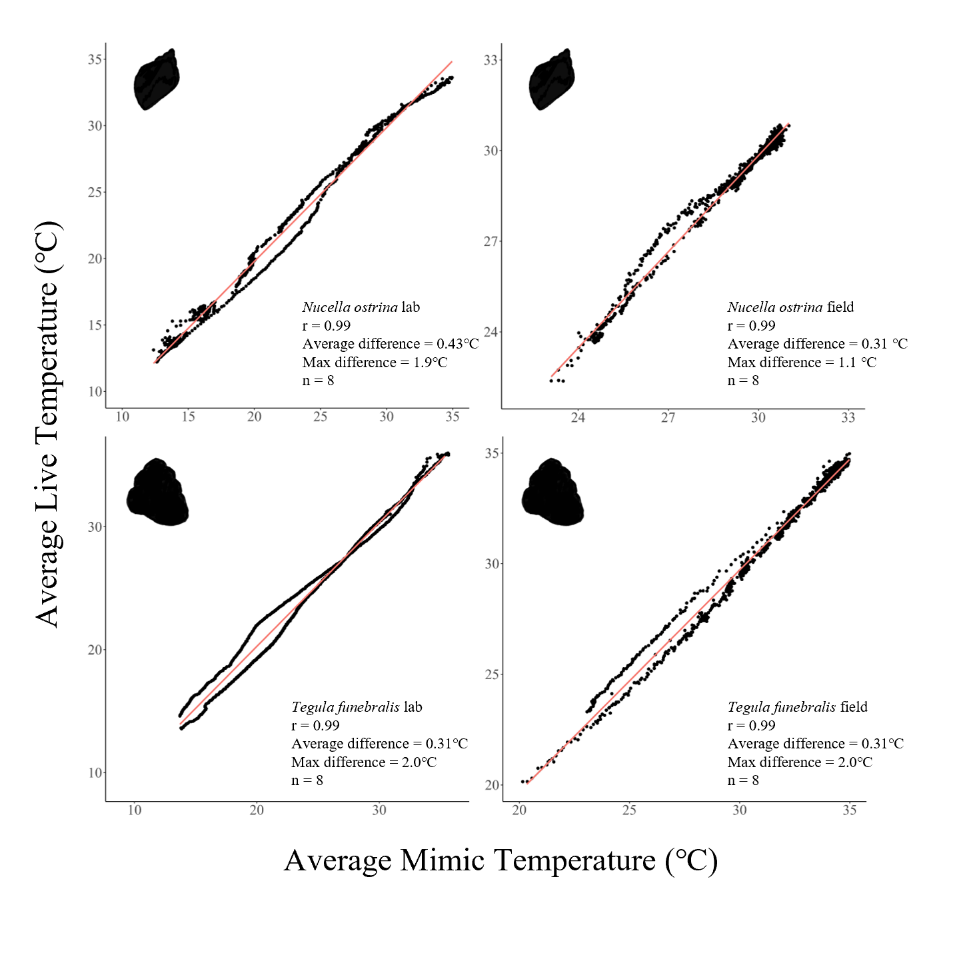


Figure S2B: The relationship between biomimic temperatures and live animal temperatures from both indoor heat lamp trials (left column) and outdoor field trials (right column) is shown for the two intermediate species. Eight live animals and 8 mimics were placed together either in the laboratory or in the field with thermocouple wires embedded in both. Temperatures were taken every 5 seconds over the course of 2 hours in the lab and 1 hour in the field. Points represent the average temperature of 8 mimics and 8 live animals at a given time point. The red line indicates a 1:1 relationship, so points falling below the line indicate that the biomimic was cooler than the live animal.


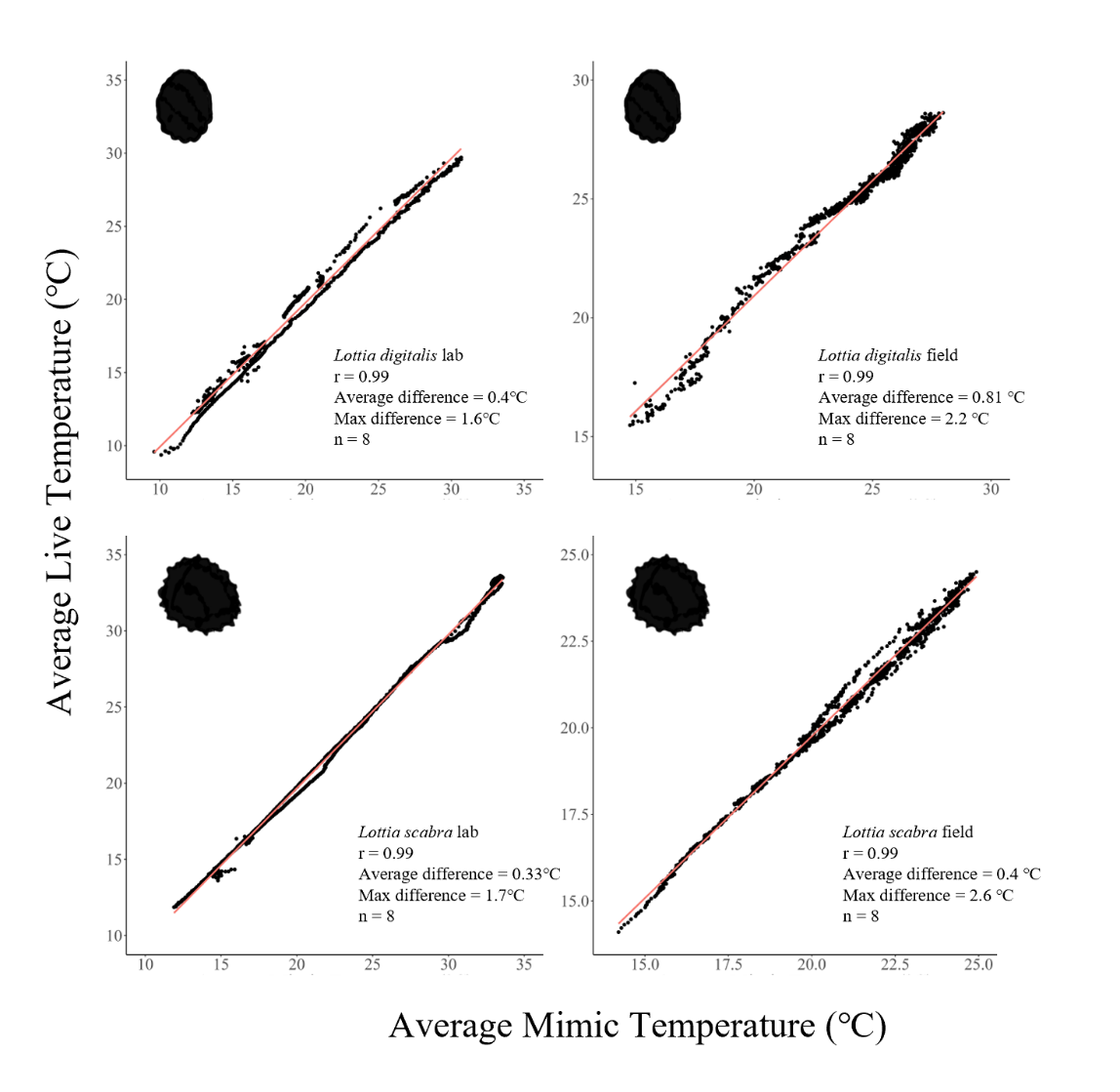


Figure S2C: The relationship between biomimic temperatures and live animal temperatures from both indoor heat lamp trials (left column) and outdoor field trials (right column) is shown for the two slow-moving species. Eight live animals and 8 mimics were placed together either in the laboratory or in the field with thermocouple wires embedded in both. Temperatures were taken every 5 seconds over the course of 2 hours in the lab and 1 hour in the field. Points represent the average temperature of 8 mimics and 8 live animals at a given time point. The red line indicates a 1:1 relationship, so points falling below the line indicate that the biomimic was cooler than the live animal.


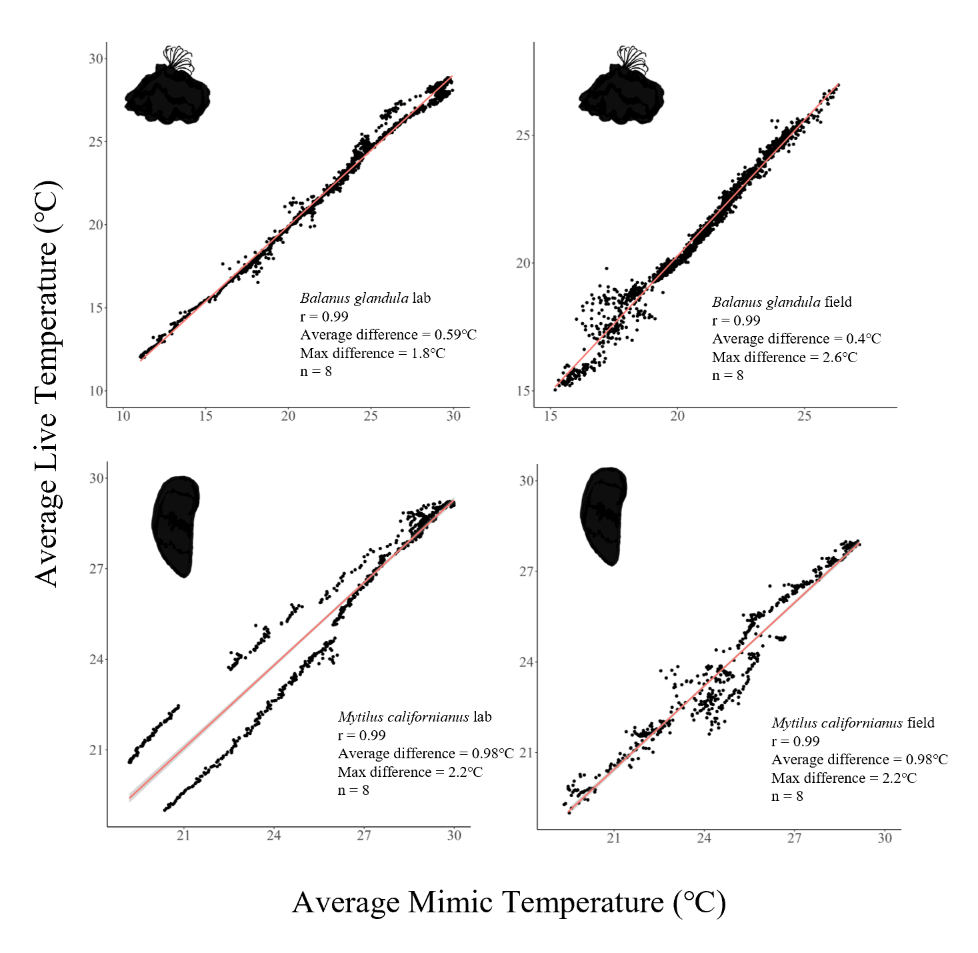


Figure S2D: The relationship between biomimic temperatures and live animal temperatures from both indoor heat lamp trials (left column) and outdoor field trials (right column) is shown for the two sessile species. Eight live animals and 8 mimics were placed together either in the laboratory or in the field with thermocouple wires embedded in both. Temperatures were taken every 5 seconds over the course of 2 hours in the lab and 1 hour in the field. Points represent the average temperature of 8 mimics and 8 live animals at a given time point. The red line indicates a 1:1 relationship, so points falling below the line indicate that the biomimic was cooler than the live animal.

Supplement 3: Results for respiration trials by species. Asterisks symbolize significance α < 0.05.

*Li. occidentalis* Tukey pairwise comparison results

| **Temperature comparison** | **Estimate** | **Std. Error** | **df** | **z value** | **Pr(>\|z\|)** |
| --- | --- | --- | --- | --- | --- |
| 14.5 - 26.1C | 21.72 | 124.57 | 11 | 168.02 | 0.99 |
| 14.5 - 31.3C | 6.43 | 97.01 | 11 | 109.88 | 1.00 |
| 14.5 - 35.4C | 70.87 | 32.58 | 11 | 174.31 | 0.31 |
| 14.5 - 38.6C | 0.68 | 104.12 | 11 | 102.77 | 1.00 |
| 26.1 - 31.3C | 15.29 | 161.58 | 11 | 131.01 | 1.00 |
| 26.1 - 35.4C | 49.15 | 97.15 | 11 | 195.44 | 0.87 |
| 26.1 - 38.6C | 22.40 | 168.69 | 11 | 123.90 | 0.99 |
| 33.3 -35.4C | 64.44 | 39.01 | 11 | 167.88 | 0.40 |
| 31.3 - 38.6C | 7.11 | 110.55 | 11 | 96.34 | 1.00 |
| 35.4 - 38.6 C | 71.54 | 174.99 | 11 | 31.90 | 0.30 |

*P. crassipes* Tukey pairwise comparison results

| **Temperature comparison** | **Estimate** | **Std. Error** | **df** | **z value** | **Pr(>\|z\|)** |
| --- | --- | --- | --- | --- | --- |
| 14.5-20.1C | 66.4 | 64.29 | 11 | 1.03 | 0.93 |
| 14.5 -26.4C | 86.09 | 46.8 | 11 | 1.84 | 0.47 |
| 14.5 - 31.7C | 160.47 | 115.04 | 11 | 1.40 | 0.77 |
| 14.5 - 35C | 276.29 | 189.54 | 11 | 1.46 | 0.73 |
| 14.5 - 36.9C | 324.52 | 145.02 | 11 | 2.24 | 0.24 |
| 14.5 - 37.4C | -30.64 | 57.01 | 11 | -0.54 | 1.00 |
| 20.1 - 26.4C | 19.69 | 69.1 | 11 | 0.29 | 1.00 |
| 20.1-31.7C | 94.07 | 125.78 | 11 | 0.75 | 0.99 |
| 20.1 - 35C | 209.89 | 196.24 | 11 | 1.07 | 0.92 |
| 20.1-36.9C | 258.13 | 153.67 | 11 | 1.68 | 0.58 |
| 20.1 -37.4C | -97.03 | 76.39 | 11 | -1.27 | 0.84 |
| 26.4-31.7C | 74.38 | 117.8 | 11 | 0.63 | 1.00 |
| 26.1-35C | 190.2 | 191.23 | 11 | 1.00 | 0.94 |
| 26.1-36.9C | 238.43 | 147.22 | 11 | 1.62 | 0.62 |
| 26.1-37.4C | -116.72 | 62.39 | 11 | -1.87 | 0.45 |
| 31.7-35C | 115.82 | 218.21 | 11 | 0.53 | 1.00 |
| 31.7-36.9C | 164.05 | 180.88 | 11 | 0.91 | 0.96 |
| 31.7-37.4C | -191.11 | 122.22 | 11 | -1.56 | 0.66 |
| 35 - 36.9C | 48.24 | 235.39 | 11 | 0.21 | 1.00 |
| 35 - 37.4C | -306.92 | 193.98 | 11 | -1.58 | 0.65 |
| 36.9 - 37.4C | -355.16 | 150.77 | 11 | -2.36 | 0.18 |

*N. ostrina* Tukey pairwise comparison results

| **Temperature comparison** | **Estimate** | **Std. Error** | **df** | **z value** | **Pr(>\|z\|)** |
| --- | --- | --- | --- | --- | --- |
| 14.5 - 26.1C | 1.98 | 4.55 | 11 | 0.43 | 0.99 |
| 14.5 - 32.4C | 24.90 | 8.31 | 11 | 3.00 | 0.02* |
| 14.5 - 34.2C | 53.41 | 18.01 | 11 | 2.96 | 0.02* |
| 14.5 - 39.1C | 48.34 | 16.85 | 11 | 2.87 | 0.03* |
| 26.1 - 32.4C | 22.94 | 8.07 | 11 | 2.84 | 0.03* |
| 26.1 - 34.2C | 51.43 | 17.91 | 11 | 2.87 | 0.03* |
| 26.1 - 39.1C | 46.37 | 16.74 | 11 | 2.77 | 0.04* |
| 32.4 -34.2C | 28.49 | 19.21 | 11 | 1.48 | 0.53 |
| 32.4 - 39.1C | 23.43 | 18.12 | 11 | 1.29 | 0.66 |
| 34.2 - 39.1C | -5.07 | 24.17 | 11 | -0.21 | 1.00 |

*T. funebralis* Tukey pairwise comparison results

| **Temperature comparison** | **Estimate** | **Std. Error** | **df** | **z value** | **Pr(>\|z\|)** |
| --- | --- | --- | --- | --- | --- |
| 14 - 26C | -0.83 | 12.49 | 11 | -0.07 | 1 |
| 14 - 32C | 7.73 | 13.72 | 11 | 0.56 | 0.98 |
| 14 - 40C | 17.92 | 15.90 | 11 | 1.13 | 0.78 |
| 14- 44C | -13.19 | 11.36 | 11 | -1.16 | 0.76 |
| 26 - 32C | 8.57 | 11.04 | 11 | 0.78 | 0.93 |
| 26 - 40C | 18.75 | 13.65 | 11 | 1.37 | 0.63 |
| 26 - 44C | -12.36 | 7.93 | 11 | -1.56 | 0.51 |
| 32 - 40C | 10.19 | 14.78 | 11 | 0.69 | 0.96 |
| 32 - 44C | -20.93 | 9.74 | 11 | -2.15 | 0.19 |
| 40 - 44C | -31.11 | 12.63 | 11 | -2.46 | 0.09 |

Supplement 4

Table S4: Chi-squared tests of independence comparing the distribution of live animals when compared to the amount of each habitat available in sampled transects.

| **Species** | **χ2** | **df** | ***P-value*** |
| --- | --- | --- | --- |
| *Li. occidentalis* | 28.52 | 3 | <<0.01 |
| *P. crassipes* | 366.55 | 3 | <<0.01 |
| *T. funebralis* | 58.42 | 3 | <<0.01 |
| *N. ostrina* | 28.22 | 3 | <<0.01 |
| *L. digitalis* | 58.12 | 3 | <<0.01 |
| *L. scabra* | 63.23 | 3 | <<0.01 |
| *M. californianus* | 11.59 | 3 | 0.01 |
| *B. glandula* | 75.28 | 3 | <<0.01 |


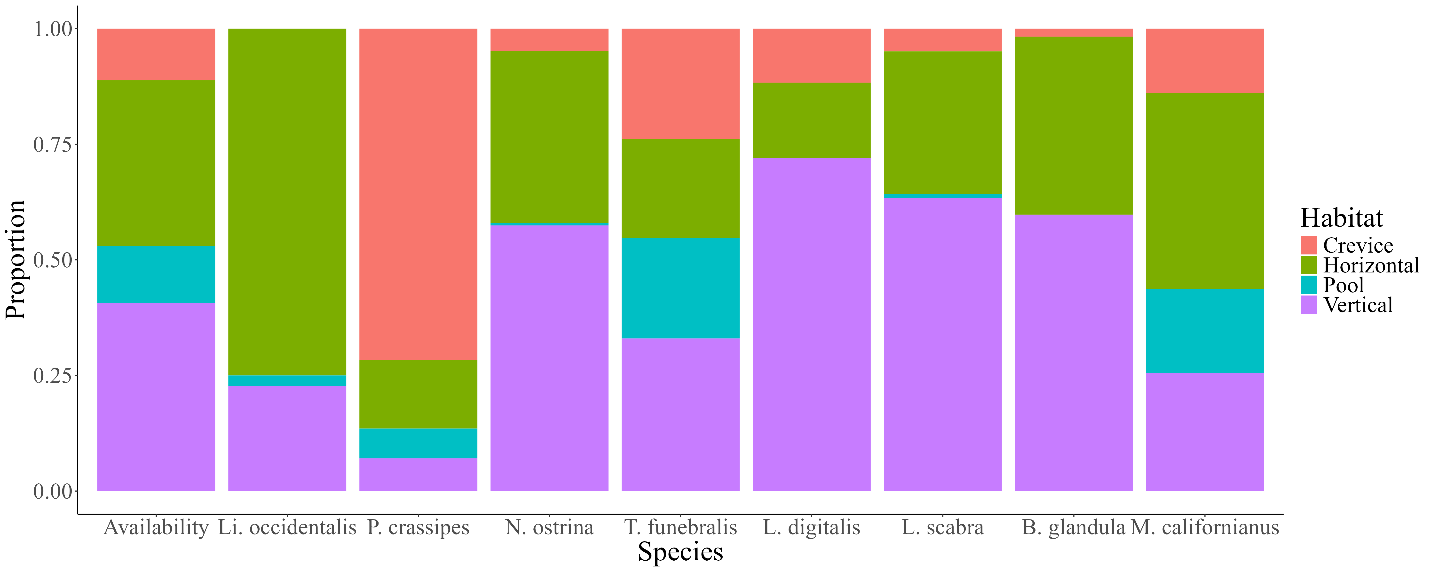


Figure S4: The proportion of habitats available at the field sites (“Availability”) in comparison to where our study species were found. At the sites, we quantified the number of available habitats (crevices, horizontal surfaces, vertical surface, and pools) using a 0.25m^2^ quadrat on both sides of transects. Species’ habitat selection was recorded along the transects during the body temperature surveys.
