# Supplementary material for: The Role of Mobility in Intertidal Invertebrates’ Responses to Thermal Stress
Source: Integr Comp Biol. 2025 Jun 3;65(4):812–21. doi: 10.1093/icb/icaf078 (PMC12530186; doi:10.1093/icb/icaf078)
Supplement: icaf078_Supplemental_Files [file icaf078_supplemental_files.zip › icb-2025-0113-File008.docx]

Supplement 3: Results for respiration trials by species. Asterisks symbolize significance α < 0.05.

A) *Li. occidentalis* Tukey pairwise comparison results

| **Temperature comparison** | **Estimate** | **Std. Error** | **df** | **z value** | **Pr(>\|z\|)** |
| --- | --- | --- | --- | --- | --- |
| 14.5 - 26.1C | 21.72 | 124.57 | 11 | 168.02 | 0.99 |
| 14.5 - 31.3C | 6.43 | 97.01 | 11 | 109.88 | 1.00 |
| 14.5 - 35.4C | 70.87 | 32.58 | 11 | 174.31 | 0.31 |
| 14.5 - 38.6C | 0.68 | 104.12 | 11 | 102.77 | 1.00 |
| 26.1 - 31.3C | 15.29 | 161.58 | 11 | 131.01 | 1.00 |
| 26.1 - 35.4C | 49.15 | 97.15 | 11 | 195.44 | 0.87 |
| 26.1 - 38.6C | 22.40 | 168.69 | 11 | 123.90 | 0.99 |
| 33.3 -35.4C | 64.44 | 39.01 | 11 | 167.88 | 0.40 |
| 31.3 - 38.6C | 7.11 | 110.55 | 11 | 96.34 | 1.00 |
| 35.4 - 38.6 C | 71.54 | 174.99 | 11 | 31.90 | 0.30 |

B) *P. crassipes* Tukey pairwise comparison results

| **Temperature comparison** | **Estimate** | **Std. Error** | **df** | **z value** | **Pr(>\|z\|)** |
| --- | --- | --- | --- | --- | --- |
| 14.5-20.1C | 66.4 | 64.29 | 11 | 1.03 | 0.93 |
| 14.5 -26.4C | 86.09 | 46.8 | 11 | 1.84 | 0.47 |
| 14.5 - 31.7C | 160.47 | 115.04 | 11 | 1.40 | 0.77 |
| 14.5 - 35C | 276.29 | 189.54 | 11 | 1.46 | 0.73 |
| 14.5 - 36.9C | 324.52 | 145.02 | 11 | 2.24 | 0.24 |
| 14.5 - 37.4C | -30.64 | 57.01 | 11 | -0.54 | 1.00 |
| 20.1 - 26.4C | 19.69 | 69.1 | 11 | 0.29 | 1.00 |
| 20.1-31.7C | 94.07 | 125.78 | 11 | 0.75 | 0.99 |
| 20.1 - 35C | 209.89 | 196.24 | 11 | 1.07 | 0.92 |
| 20.1-36.9C | 258.13 | 153.67 | 11 | 1.68 | 0.58 |
| 20.1 -37.4C | -97.03 | 76.39 | 11 | -1.27 | 0.84 |
| 26.4-31.7C | 74.38 | 117.8 | 11 | 0.63 | 1.00 |
| 26.1-35C | 190.2 | 191.23 | 11 | 1.00 | 0.94 |
| 26.1-36.9C | 238.43 | 147.22 | 11 | 1.62 | 0.62 |
| 26.1-37.4C | -116.72 | 62.39 | 11 | -1.87 | 0.45 |
| 31.7-35C | 115.82 | 218.21 | 11 | 0.53 | 1.00 |
| 31.7-36.9C | 164.05 | 180.88 | 11 | 0.91 | 0.96 |
| 31.7-37.4C | -191.11 | 122.22 | 11 | -1.56 | 0.66 |
| 35 - 36.9C | 48.24 | 235.39 | 11 | 0.21 | 1.00 |
| 35 - 37.4C | -306.92 | 193.98 | 11 | -1.58 | 0.65 |
| 36.9 - 37.4C | -355.16 | 150.77 | 11 | -2.36 | 0.18 |

C) *N. ostrina* Tukey pairwise comparison results

| **Temperature comparison** | **Estimate** | **Std. Error** | **df** | **z value** | **Pr(>\|z\|)** |
| --- | --- | --- | --- | --- | --- |
| 14.5 - 26.1C | 1.98 | 4.55 | 11 | 0.43 | 0.99 |
| 14.5 - 32.4C | 24.90 | 8.31 | 11 | 3.00 | 0.02* |
| 14.5 - 34.2C | 53.41 | 18.01 | 11 | 2.96 | 0.02* |
| 14.5 - 39.1C | 48.34 | 16.85 | 11 | 2.87 | 0.03* |
| 26.1 - 32.4C | 22.94 | 8.07 | 11 | 2.84 | 0.03* |
| 26.1 - 34.2C | 51.43 | 17.91 | 11 | 2.87 | 0.03* |
| 26.1 - 39.1C | 46.37 | 16.74 | 11 | 2.77 | 0.04* |
| 32.4 -34.2C | 28.49 | 19.21 | 11 | 1.48 | 0.53 |
| 32.4 - 39.1C | 23.43 | 18.12 | 11 | 1.29 | 0.66 |
| 34.2 - 39.1C | -5.07 | 24.17 | 11 | -0.21 | 1.00 |

D) *T. funebralis* Tukey pairwise comparison results

| **Temperature comparison** | **Estimate** | **Std. Error** | **df** | **z value** | **Pr(>\|z\|)** |
| --- | --- | --- | --- | --- | --- |
| 14 - 26C | -0.83 | 12.49 | 11 | -0.07 | 1 |
| 14 - 32C | 7.73 | 13.72 | 11 | 0.56 | 0.98 |
| 14 - 40C | 17.92 | 15.90 | 11 | 1.13 | 0.78 |
| 14- 44C | -13.19 | 11.36 | 11 | -1.16 | 0.76 |
| 26 - 32C | 8.57 | 11.04 | 11 | 0.78 | 0.93 |
| 26 - 40C | 18.75 | 13.65 | 11 | 1.37 | 0.63 |
| 26 - 44C | -12.36 | 7.93 | 11 | -1.56 | 0.51 |
| 32 - 40C | 10.19 | 14.78 | 11 | 0.69 | 0.96 |
| 32 - 44C | -20.93 | 9.74 | 11 | -2.15 | 0.19 |
| 40 - 44C | -31.11 | 12.63 | 11 | -2.46 | 0.09 |

Supplement 4

Table S4: Chi-squared tests of independence comparing the distribution of live animals when compared to the amount of each habitat available in sampled transects.

| **Species** | **χ2** | **df** | ***P-value*** |
| --- | --- | --- | --- |
| *Li. occidentalis* | 28.52 | 3 | <<0.01 |
| *P. crassipes* | 366.55 | 3 | <<0.01 |
| *T. funebralis* | 58.42 | 3 | <<0.01 |
| *N. ostrina* | 28.22 | 3 | <<0.01 |
| *L. digitalis* | 58.12 | 3 | <<0.01 |
| *L. scabra* | 63.23 | 3 | <<0.01 |
| *M. californianus* | 11.59 | 3 | 0.01 |
| *B. glandula* | 75.28 | 3 | <<0.01 |
